# Mid- to long-term functional outcome and return to sport after elbow dislocation in bouldering: a clinical retrospective cohort study

**DOI:** 10.1007/s00402-024-05397-0

**Published:** 2024-06-13

**Authors:** M. Müller, S. Pedersen, O. Mair, V. Twardy, S. Siebenlist, P. Biberthaler, I.J. Banke

**Affiliations:** 1 Department of Trauma Surgery, Klinikum rechts der Isar, Technical University of Munich, Ismaninger Strasse 22, 81675 Munich, Germany; 2 Clinic of Orthopedics and Sports Orthopedics, Klinikum rechts der Isar, Technical University of Munich, Munich, Germany; 3 Department of Sports Orthopedics, Klinikum rechts der Isar, Technical University of Munich, Munich, Germany

**Keywords:** Return to sports, Elbow trauma, Traumatic elbow dislocation, Indoor rock climbing, Bouldering, ESAS score

## Abstract

**Introduction:**

Traumatic elbow dislocations are among the most common injuries in sport climbing. They occur most frequently in bouldering (a climbing discipline with strong upward trend often performed indoors) due to the typical low-height backward fall into crashpads. There is still no data about the functional outcome and return to sport of this typical bouldering injury.

**Materials and methods:**

All Patients with elbow dislocations due to a bouldering associated fall between 2011 and 2020 were identified retrospectively in our level I trauma centre. Trauma mechanisms, injury types and therapies were obtained. Follow-up was performed with an online questionnaire including sports-related effects, return to sport and the Elbow Self-Assessment Score (ESAS).

**Results:**

30 patients with elbow dislocations after bouldering accidents were identified. In 22 (73.3%) patients the injury was a simple dislocation. The questionnaire was completed by 20 patients. The leading mechanism was a low-height fall into crashpads. Surgical procedures were performed in every second patient. 18 patients (90%) reported return to bouldering after 4.7 ± 2.1 months. 12 patients (66.7%) regained their pre-injury level. Mid-/Long-term follow-up (mean 105 ± 37.5 months) showed excellent results in ESAS score (97.2 ± 3.9 points). Persistent limited range of motion or instability was reported by only 3 patients (15%).

**Conclusion:**

Most athletes are able to return to bouldering but only two thirds regain their pre-injury performance level in this demanding upper-extremity sport. The unique low-height trauma mechanism may create a false sense of security. Specific awareness and safety features should be placed for climbing athletes to reduce elbow injuries.

**Supplementary Information:**

The online version contains supplementary material available at 10.1007/s00402-024-05397-0.

## Introduction

Indoor rock climbing (bouldering) is a modern discipline originated from traditional rock climbing. It shows a strong upward trend among both non-competitive and competitive athletes, also reflected by its debut in the 2020 Tokyo Olympic Games. Compared to normal climbing bouldering is generally thought to be a rather harmless sport due to the low-height making a climbing rope useless.

Generally, elbow dislocations heal with good results, but severe dislocations are at risk of chronic instability and reduced function [1]. While elbow dislocations play a minor role in classic sport climbing [2, 3], they are seen frequently in the increasingly popular bouldering [4]. Principally the elbow is the third most affected anatomic area in bouldering injuries following ankle and knee [5]. The highly sport-specific trauma mechanism is a backward fall from low-height (1–5 m) dipping the arm with extended elbow into a soft foam crashpad. There are ongoing discussions about the exact biomechanical sequence of the dislocation [6, 7]. The most common model goes back to the studies of O’Driscoll and includes a combination of axial loading, valgus stress and supination, leading the elbow to dislocation [8].

Most elbow dislocations are so called “simple” dislocations without relevant bony lesions [9]. The most common treatment method is closed reduction and conservative treatment, leading to excellent results [10, 11]. Complex dislocations are injuries with concomitant lesions of the proximal radius, ulna or proximal humerus [9]. These lesions as well as all unstable injuries with tendency of redislocation should undergo surgical therapy achieving also good clinical results [12].

The steadily growing number of bouldering athletes will lead to an increasing number of elbow dislocations in the future. There is literature about the functional outcome after elbow dislocations in common [12, 13]. However, there is no sufficient data yet on the effects of these injuries on functional outcome, return to sports and future performance for bouldering athletes. Since bouldering involves high forces on the elbow, it is a relevant question whether boulder climbers will return to their pre-injury level. The purpose of this study was to describe the effects of these injuries on the patient’s functionality and athletic performance. Awareness should be given for athletes as well as indoor climbing centres to reduce elbow injuries to the best extend making bouldering as safe as possible.

## Methods

### Patients and study design

The study protocol was approved by the local ethics committee (Study number 309/20 S). All bouldering-associated elbow dislocations treated at our level I university trauma centre between 2011 and 2020 were identified. Case-related injury information was captured from the electronic documentation system. Injured side, type of injury, mode of repositioning, necessity and type of surgical treatment, and the need of inpatient admission were taken into account. In order to classify the injury patterns as precisely as possible, conventional x-ray, computer tomography and, if available MRI were analysed for assessment of the involved injured structures.

### Diagnostic and treatment

Treatment decision was based on our standardized treatment algorithm. If the dislocation has not been reduced prior to the presentation to the emergency department it will then be done under analgosedation. All patients with an elbow dislocation received x-ray diagnostics. If the x-ray showed a bony lesion, CT imaging was additionally performed. In addition, MRI imaging was initiated to assess potential cartilaginous lesions, the integrity of the extensor and flexor muscles origins and the collateral ligaments. According to our clinical routine treatment algorithm for elbow dislocations an initial dynamic examination of elbows stability is carried out immediately after reduction under analgosedation. This allows the primary stability to be tested, as the muscular joint tension is reduced by the analgosedation. Patients are then clinically re-evaluated at an interval of 3–5 days in order to repeat the dynamic stability test with the patient awake and using an x-ray image converter. Simple dislocations without tendency for further dislocation and with congruent joint alignment within the functional arc between 30° and 130° were treated conservatively. Joints were defined as incongruent, when presenting a positive “drop sign” in the lateral x-ray diagnostics. For patients with persisting instability, relevant fractures and ruptures involving the insertions of the flexor- and extensor-muscles surgical treatment is recommended. Unstable and incongruent joints or dislocated fractures were treated immediately surgically. Treatment of choice was reconstruction of the affected ligaments and muscle origins using suture anchors. If the LUCL was involved, radial ligament stabilization was started. This was followed by re-evaluation and examination of the medial ligamentous complex in the event of persistent instability, surgical stabilization with suture anchors was then also performed. A solely medial stabilisation was performed if preoperative diagnostics showed an isolated medial instability. Dislocated fractures were treated with open reduction and internal fixation. The strategy for the osteosynthetic treatment of articular fractures was determined on the basis of preoperative imaging. The aftercare regimen includes only a short immobilization in a plaster, followed by early movement practice in a hinged brace.

**Fig. 1:**
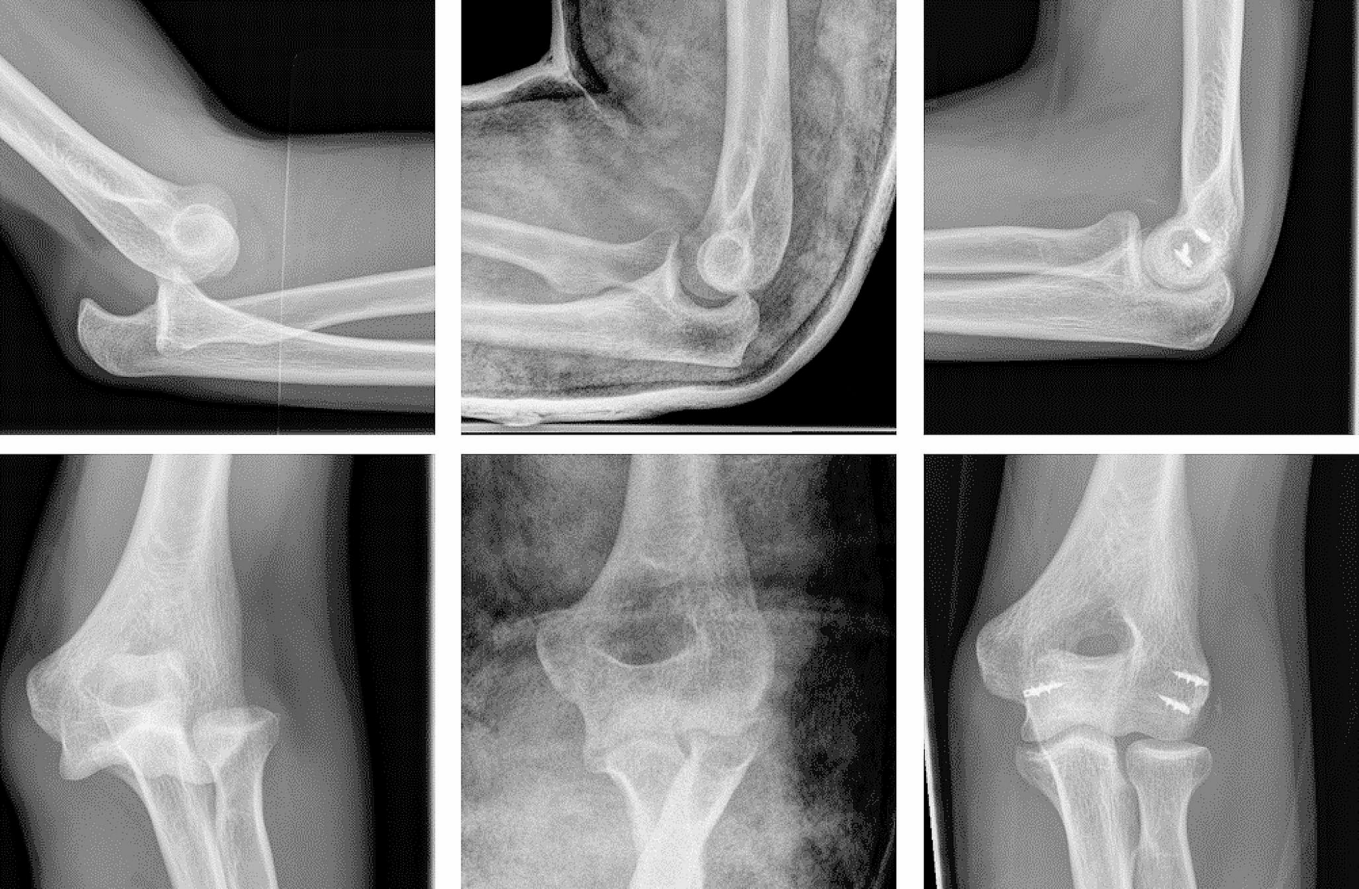
x-ray diagnostics: **left** acute posterior elbow dislocation after fall in bouldering; **middle**: drop sign indicating persisting instability after reduction; **right**: postoperative results after capsular-ligament reconstruction of the radial and ulnar collateral ligament and the common extensor origin

Figure 1 shows exemplary radiographs of acute dislocations prior to and after reduction as well as the postoperative results.

### Follow up

All patients were invited to participate in a follow-up assessment using an online questionnaire. The questionnaire included subjective performance level before and after the accident on the established Fountainblaeu scale [14], as well as on return to sport. The follow-up intervals are defined by consensus as mid-term (minimum 60 months) and long-term (minimum 150 months) [15]. Functional outcome was measured by using the ESAS (elbow self-assessment score) PROM, a validated patient-based questionnaire that contains items on functionality and pain and also includes image-based items on range of motion [16].

### Statistics

Frequencies of variables were specified with the number and the percentage share. For bivariate analyses continuous variables were described with mean ± standard deviation. Binary variables were compared with percentages in cross-tables. Pearson’s-Chi-Square test was used to validate significance. Continuous variables were compared using Mann-Whitney-U Test. The level of significance was defined as *p* < 0.05. Statistics were calculated using SPSS (IBM SPSS Statistics for Windows, Version 26.0; Armonk, N.Y., USA).

## Results

### Epidemiological data

A total of 30 patients were identified who had sustained an elbow dislocation in the course of a bouldering trauma. 17 patients (56.7%) were male and 13 (43.3%) were female. The average patient age was 30.2 ± 10.3 years. The youngest patient was 17 years old and the oldest 53 years old at the time of the accident. The injuries affected the left elbow in 20 patients (66.7%) and the right elbow in 10 patients (33.3%). In 9 cases (30%), the dislocation was already reduced preclinically by an emergency doctor. In the remaining 21 patients (70%), the reduction was performed in the emergency room. Due to the localization of our clinic in an urban area, the accidents occurred particularly in indoor bouldering gyms.

### Injury patterns

Height of fall was 1–2 m (*n* = 6; 30%), 2–3 m (*n* = 7; 35%) and 3–4 m (*n* = 7; 35%). As reported by all patients common trauma mechanism was a backward fall dipping the arm with extended elbow into a soft foam crashpad. All dislocations were posterior. Radiological diagnostics revealed that 23 patients (73.3%) had simple elbow dislocations. In 7 patients (23%) concomitant bony injury was present with 4 patients (13.3.0%) showing a radial head fracture (Mason classification: 2x type I, 2x type II) and 4 patients (10%) an Osborne Coterill lesion. In 6 patients (20%) direct inpatient admission was necessary for early surgical treatment. The detailed injury patterns for the follow-up group of 20 patients that participated in the functional outcome evaluation is demonstrated in Table 1. No concomitant neurovascular injuries were described in any of the patients prior to or after the reductions.

### Follow up and functional outcome

All 30 patients were invited to participate in the follow-up investigation via online questionnaire. Of these, 20 patients completed the survey in full, resulting in a response rate of 66.7%. Mean follow up was 105 ± 37.5 months. Minimal follow up was 38 months and maximum was 159 months. Among the patients followed up, the average age was 29 ± 9.9 years. 10 women (50%) and 10 men (50%) answered the questionnaire.

**Fig. 2:**
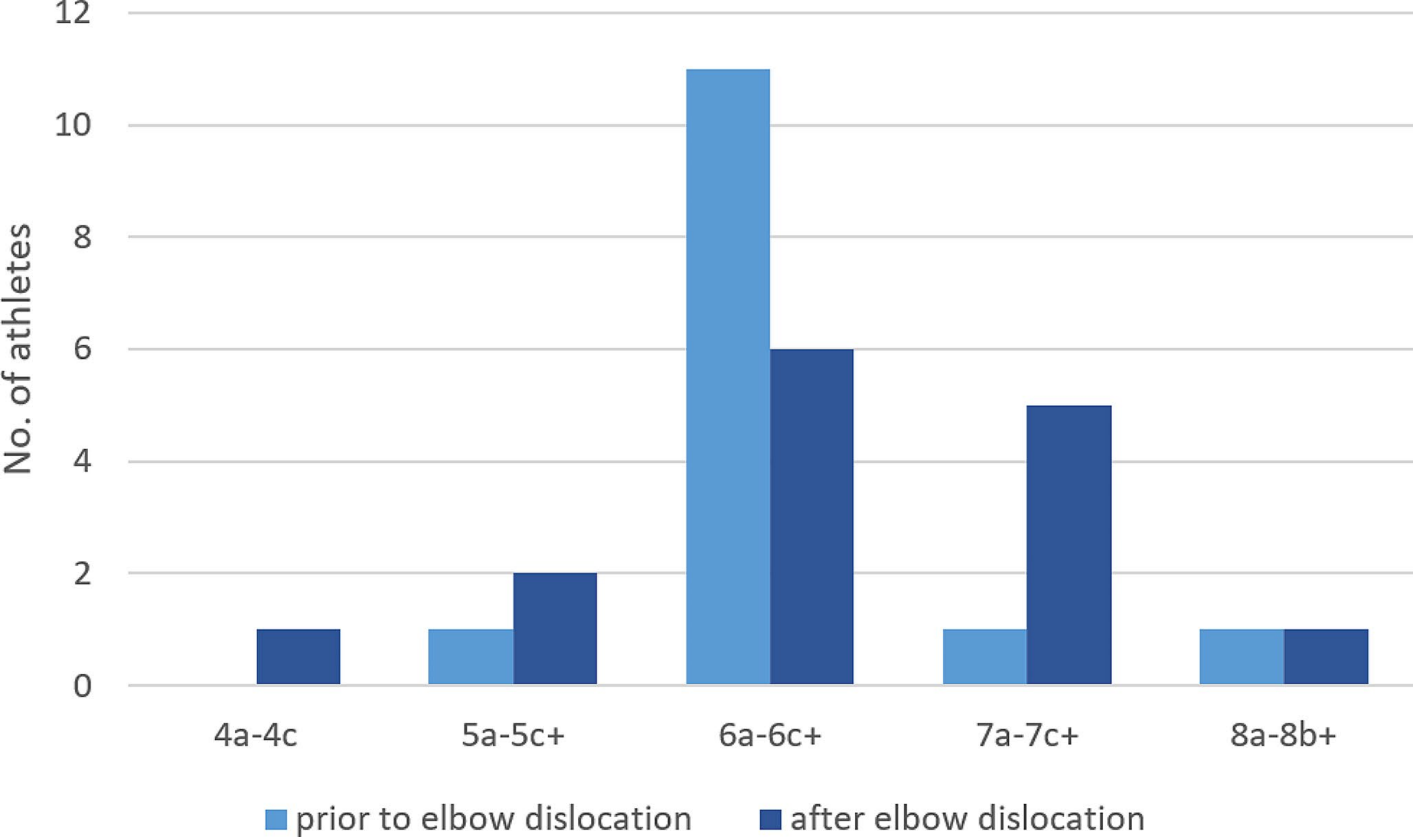
Frequencies of levels of performance on the Fontainebleau scale prior to and after the elbow dislocation

18 patients (90%) reported, that they were able to return to the bouldering centres. The mean time to return to sport was 4.7 ± 2.1 months (minimum 1 month, maximum 9 months). Two patients quit bouldering after the elbow dislocation. Both stated fear of further injury. Among the athletes who returned to sport, 13 (72.2%) reported that their performance had been limited due to fear of falling again. 12 (66.7%) of the returning athletes were able to reach or even exceed their initial performance level. As measured by self-reported performance on the Fontainebleau (Fb) scale, 4 patients (22.2%) improved their performance, 10 (55.5%) reported that they reached the previous level, and 4 (22.2%) reported a lower level of performance on the Fb grading. A graphical overview of the distribution of the performance levels is shown in Fig. 2.

The mean elbow self-assessment score (ESAS) was 97.2 ± 3.9 points. In the item for range of motion in the ESAS questionnaire three patients (15%) reported the inability for full extension in the elbow. The subjective feeling of persistent instability in the elbow joint was reported by 3 patients (15%). 7 patients (35%) reported a persisting impairment during sports and other activities, but all of them only with a 1 point grading on a 10 point likert scale.

Surgical or conservative therapy was performed for 10 patients (50%) each. The operations were performed by 3 different senior surgeons specialized in upper extremity. Of those patients who were treated surgically, two patients did not return to bouldering. Among the conservatively treated patients, every patient stated that they had returned to bouldering. This difference was not statistically significant (*p* = 0.237). There was also no significant difference in the time to return to sport between the patients who underwent surgery (5 ± 1.7 months) and those who received conservative therapy (4.5 ± 2.6 months) (*p* = 0.460). There were also no significant deviations between the ESAS scores of patients who underwent surgery (96.8 ± 4.5 points) and of those who received conservative treatment (97.6 ± 3.6 points) (*p* = 0.684).

### Patients lost to follow up

Twenty out of thirty patients completed the questionnaire. There was no significant difference in age (31.3 ± 11.4 vs. 29.7 ± 9.9 years; *p* = 0.846), gender (50% male vs. 70% male; *p* = 0.26), need for operative treatment (20% vs. 50%; *p* = 0.117) or presence of bony lesions (20% vs. 30%; *p* = 0.429) between the patients who completed the questionnaire and those who did not.

**Table 1:** Epidemiologics and injury patterns for the 20 patients, that participated in the follow up study

	*N* (%) or mean ± SD
**N total**	20
**Age [years]**	29.7 ± 9.9
**Gender**	
female	10 (50%)
male	10 (50%)
**Dislocation type**	
simple	15 (75%)
complex	5 (25%)
**Bony lesions**	
Radial head fracture	3 (15%)
Osborne cotterill lesion	2 (10%)
Ligamentous instabilities*	
MCL / flexor muscles	4 (20%)
LUCL / extensor muscles	5 (25%)
MCL and LUCL	9 (45%)

MCL = medial collateral ligament; LUCL = lateral ulnar collateral ligament; SD= standard deviation; * based on clinical

## Discussion

Elbow dislocations mostly occur during sports activities. Typical mechanisms are wrestling-, football- and, especially in children, trampoline-accidents [17, 18]. This study presents a representative cohort of patients with elbow dislocations that occurred in the context of bouldering and examines the mid- to long-term consequences on the functionality and athletic activity of climbing athletes after a mean period of nearly 9 years.

Elbow dislocations are extremely painful and traumatizing events for the patient. The severity of this experience gives the patient the impression of a serious injury with lasting consequences. The results of this study nevertheless present encouraging results in this respect as the rate of patients who return to bouldering with 90% is very high. Only two patients who underwent surgical treatment did not return to bouldering.

Geyer et al. investigated the functional outcome of operatively and conservatively treated simple elbow dislocations following various sport, daily activities and working injuries and reported a return to sport rate of even 100%. However, the authors examined general athletic ability rather than specific return to a particular high upper extremity demanding sport. Furthermore, they describe several patients who quit specific types of sports and shifted their activity profile. Olsen et al. described a collective of 18 patients after surgical stabilization of the elbow and reported 15 patients (83%) who were able to return to their previous activities [19].

Even in the field of professional sports, good healing processes have been described for elbow dislocations. In a study in the US National Football League (NFL), 75.8% of athletes were able to return to competitive sports in the same season [20]. These professional football players returned to play at a mean of 26 days, whereas the mostly recreational bouldering athletes of our study reported a mean break of 4.7 months, which can be explained by the higher performance pressure in the NFL and the higher forces on the upper extremities in bouldering.

Some physicians recommend that elbow dislocations should only be reduced in the emergency room and after X-ray diagnostics have been performed to exclude a fracture in the area of the adjoining bones [21]. However, in this study, 9 of 30 patients (70%) had already been reduced prehospital by the emergency physician. Skelley et al. describe the clinical management of various in-game joint dislocations during athletic activities. They mention poorer reduction conditions due to lack of skilled personnel and limited opportunity for anaesthesia. However, with appropriate skills and knowledge about the anatomy, reduction can be performed on the field to spare the patient pain and avoid neurovascular compromise [22]. Other authors share this opinion that reduction can already be performed before radiographic diagnostic [23]. Common reduction techniques usually involve mild valgus stress at the outset, followed by translation and longitudinal traction [21, 24]. Even in the presence of a fracture of the radial head or coronoid process, this maneuver would not differ. In this study, no negative consequences were observed after preclinical reduction.

Mid- to Long-term functional outcomes were assessed using the ESAS PROM. The results were excellent with an average of 97.2 points. Anakwe et al. studied simple elbow dislocations with a follow-up interval of similar lenght. Functionality was assessed using the DASH score and Oxford elbow score. Both scores were used for validation of the ESAS and can therefore be used for comparison. Anakwe also describes good results with an Oxford elbow score of 90.3 and DASH score of 6.7 [13]. This correlates with our results. The instability described by the authors (8%) also occurs with similar frequency in our collective (15%). However, if we compare the persistent movement restrictions, 56% elbow stiffness is described by Anakwe. In the boulder-athletes of this study, only 15% reported remaining movement restrictions. One reason for this could be the recommendations for even more aggressive early functional follow-up-treatments, which are widespread nowadays [25, 26].

However, the good results in the functional scores and the high proportion of patients who continue to practice the sport must not lead to a trivialization of these injuries. Even in the long follow-up period of this study, 22% of the patients were never able to return to their previous performance level after the accident.

### Limitations

The results regarding functional outcome are based on PROMs and subjective patient assessments. Especially with regard to the range of motion and stability at the elbow, these tools cannot guarantee the precision of a physician-based follow-up examination. In addition, the long follow-up period may lead to a recall bias. The low response rate is likely to be due to the long follow-up period. However, a selection bias cannot be ruled out here either. Finally, no long-term imaging was performed to exclude possible degeneration / early osteoarthritis limiting outcome. Nevertheless, this rather small sample size represents the largest known patient population for this specific injury in bouldering and provides important information about the patient’s perspective after this trauma.

## Conclusion

This study hopefully provides valuable information for bouldering athletes who sustain an elbow dislocation. Precise clinical evaluation combined with high quality radiological imaging and a staged treatment algorithm ensure satisfying results. Despite mid-/long term functional results are excellent and the vast majority can return to bouldering sports both after conservative or surgical treatment, more awareness should be placed on improved safety features.

### Electronic supplementary material

Below is the link to the electronic supplementary material.

Supplementary Material 1
