# Supplementary material for: Mid- to long-term functional outcome and return to sport after elbow dislocation in bouldering: a clinical retrospective cohort study
Source: Arch Orthop Trauma Surg. 2024 Jun 13;144(7):3145–51. doi: 10.1007/s00402-024-05397-0 (PMC11319378; doi:10.1007/s00402-024-05397-0)
Supplement: Supplementary file 1 — Supplementary Material 1 [file 402_2024_5397_MOESM1_ESM.docx]

**Mid- to long-term functional outcome and return to sport after elbow dislocation in bouldering: a clinical retrospective cohort study**

*Müller M^1^, Pedersen S^2^, Mair O^1^, Twardy V^2^, Siebenlist S^3^, Biberthaler P^1^, Banke IJ^2^*

^1^ Department of Trauma Surgery, Klinikum rechts der Isar, Technical University of Munich, Germany

^2^ Clinic of Orthopedics and Sports Orthopedics, Klinikum rechts der Isar, Technical University of Munich, Germany

^3^ Department of Sports Orthopedics, Klinikum rechts der Isar, Technical University of Munich, Germany

**Conflict of interest** All other authors declare that they have no competing interests. None of the authors have received financial payments from any commercial institution in relationship with the conduction of this study and the publication of this article.

**Background information**

IB is OR instructor for Arthrex. PB is OR instructor for DepuySynthes, Zimmer Biomet, Smith & Nephew, Arthrex, Medartis and Bonesupport. IB and PB declare that no conflicts of interest arise from these relations. All other authors declare that they have no competing interests.
